# Polyyne-Enriched Extract from *Oplopanax elatus* Significantly Ameliorates the Progression of Colon Carcinogenesis in *Apc*^Min/+^ Mice

**DOI:** 10.3390/molecules22101593

**Published:** 2017-09-22

**Authors:** Xin Qiao, Wei Sun, Chongzhi Wang, Li Zhang, Ping Li, Xiaodong Wen, Jie Yang, Chunsu Yuan

**Affiliations:** 1 State Key Laboratory of Natural Medicines, China Pharmaceutical University, Nanjing 211198, China; xinqiao@stu.cpu.edu.cn (X.Q.); cpusunw6688@126.com (W.S.); zhangli@stu.cpu.edu.cn (L.Z.); liping2004@126.com (P.L.); 2 Tang Center for Herbal Medicine Research and Department of Anesthesia & Critical Care, The Pritzker School of Medicine, The University of Chicago, Chicago, IL 60601, USA; czwang@dacc.uchicago.edu (C.W.); CYuan@dacc.uchicago.edu (C.Y.)

**Keywords:** PEO, colorectal cancer, Wnt/β-catenin signaling

## Abstract

Colorectal cancer (CRC) is the third most common cancer in the world. *Oplopanax elatus* is widely used in traditional medicine. However, little is known about its pharmacological effects and bioactive compounds. We evaluated the effects of the polyyne-enriched extract from *O. elatus* (PEO) on the progression of colon carcinogenesis in *Apc*^Min/+^ mice. In addition, these effects were also investigated in HCT116 and SW480 cells. After PEO oral administration (0.2% diet) for 12 weeks, PEO significantly improved body weight changes and reduced the tumor burden and tumor multiplicity compared with the untreated mice. Meanwhile, western blot and immunohistochemistry results showed PEO significantly reduced the expression of β-catenin and cyclinD1 in both small intestine and the colon tissues compared with the untreated mice. In addition, PEO treatment significant decreased the cell viability in both HCT116 and SW480 cell lines. It also decreased the levels of β-catenin, cyclinD1, c-myc and p-GSK-3β in HCT116 and SW480 cells at 25 μM. These results indicate that PEO may have potential value in prevention of colon cancer by down-regulating Wnt-related protein.

## 1. Introduction

Colorectal cancer (CRC) is the third most commonly diagnosed cancer and the fourth leading cause of cancer deaths around the world [1]. In 2017, it is estimated that 135,430 new CRC cases and 50,260 deaths will occur in the United States [2]. The current therapeutic approaches for CRC are mainly surgery, chemotherapy, radiotherapy, and immunotherapy. However, these strategies are often limited by severe side effects, genetic mutations and dose-limiting toxicity [3,4,5,6,7]. Meanwhile, it has been demonstrated that numerous natural products have significant anticancer effects [8]. Therefore, discovering new and less toxic compounds from herbal medicine may shed new light on the development of anticancer agents, which can selectively kill cancer cells or enhance the effects of existing chemotherapeutic agents [9].

*Oplopanax elatus* (Nakai) Nakai (*O. elatus*), grows throughout eastern Asia, including northeast China, Korea and the far east of Russia [10,11]. The plant is a member of the family Araliaceae, the same family as American ginseng and is, therefore, occasionally referred to as “Alaskan ginseng” or “Pacific ginseng”, although they are in different genera [12]. It has been long used to treat many diseases such as arthritis, diabetes mellitus, rheumatism, neurasthenia, and cardiovascular diseases [13,14]. However, compared with the extensive researches on other plants in Araliaceae, such as *Ginseng*, *Eleuther ococcus* and *Aralia* [13,15], comparatively little is known about the pharmacological effects and bioactive compounds of *O. elatus*. Phytochemical studies have revealed there are many kinds of components in *O. elatus*, such as the essensial oil, saponins, phenolic glycosides and polyynes [16]. Recently, increasing evidences have suggested polyynes have significant anti-tumor activities [17]. Facarindiol (FAD), one of polyynes isolated from *Oplopanax horridus*, can inhibit tumor growth inhibiting proteasome function and increasing accumulation of ubiquitinated proteins [18]. Similarly, oplopandiol (OPD) also has the pharmacological effects of activating endoplasmic reticulum stress and regulating BH3 family proteins to promote apoptosis of colon cancer cells [18]. Our previous studies have showed FAD and OPD are two main polyynes compounds of *O. elatus* and they are rapidly absorbted in vivo [19]. Based on this background, we hypothesized that polyyne-enriched extract from *O. elatus* (PEO) could ameliorate the progression of colon carcinogenesis. In this study, we aimed to investigate whether polyyne-enriched extract from *O. elatus* can improve the progression of colon carcinogenesis in *Apc*^Min/+^ mice. In addition, these effects were also investigated in HCT116 and SW480 cells.

## 2. Results

### 2.1. Effects of the PEO in Apc^*Min/+*^ Mice

#### 2.1.1. PEO Treatment Improved the Body Weight and Stool Bleeding in *Apc*^Min/+^ Mice

**Figure 1:**
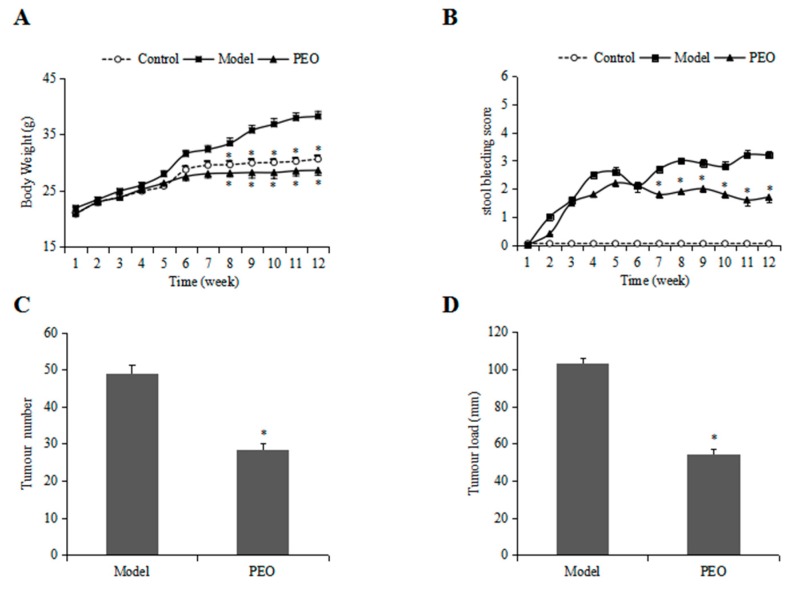
The effects of PEO on mouse body weight changes is shown in (**A**); The PEO attenuated stool bleeding score in *Apc*^Min/+^ mice (**B**); PEO treatment reduced tumor counts (**C**) and tumor load (**D**) in *Apc*^Min/+^ mice. (Data represent the means ± SE of independent experiments. * *p* < 0.05 relative to model group).

The body weight changes in different experimental groups are shown in Figure 1A. Compared with the control group, the model group had significant weight gain beginning on 6 week whereas PEO groups significantly reduced the body weight gain. In addition, the model group gradually had gross bleeding while these bleeding symptoms were greatly improved in the PEO group (Figure 1B), suggesting the reduced pathological problems.

#### 2.1.2. PEO Feeding Prevents Intestinal Tumorigenesis in *Apc*^Min/+^ Mice

**Figure 2:**
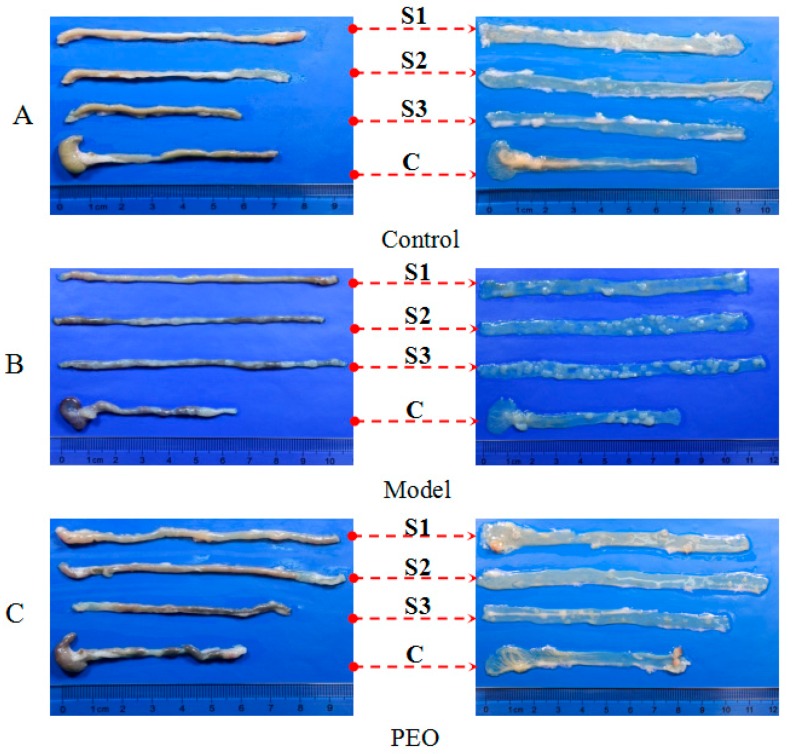
The appearance and morphology of the proximal (S1), middle (S2) and distal (S3) of the small intestine and colon (**C**). (**A**) Control group; (**B**) Model group; (**C**) PEO (0.2% diet) group.

The morphological observation results showed that no adenomatoid lesions in the control group (Figure 2A), while in the model group there were more than 40 adenomatoid lesions in the whole intestine (Figure 1C and Figure 2B). This finding is comparable with that of previous publications in *Apc*^Min/+^ mice [20]. The tumors in mice treated with PEO fortified diet were visibly less than those observed in mice treated with high fat diet (Figure 1C and Figure 2B,C). In addition, the reduction in overall tumor burden was obvious in mice treated with PEO (Figure 1D).

HE staining was carried out to observe and characterize the histological alterations. As shown in Figure S1A,B, the model group showed severe crypt dysplasia, adenomas, and adenocarcinomas in the colon compared with normal group. Inflammation, leukocyte infiltration into the lumen, nuclear hyperchromasia, nuclear mitosis, and increased nucleus-to cytoplasm ratio were observed in the adenocarcinomas of *Apc*^Min/+^ mice. However, administering PEO greatly reduced these dysplastic changes in both intestine and colon slides (Figure S1C). Taken together, these results demonstrate that dietary feeding of PEO effectively suppresses tumor growth in *Apc*^Min/+^ mice. 

#### 2.1.3. PEO Decreased the Expression of β-Catenin and CyclinD1 in *Apc*^Min/+^ Mice

**Figure 3:**
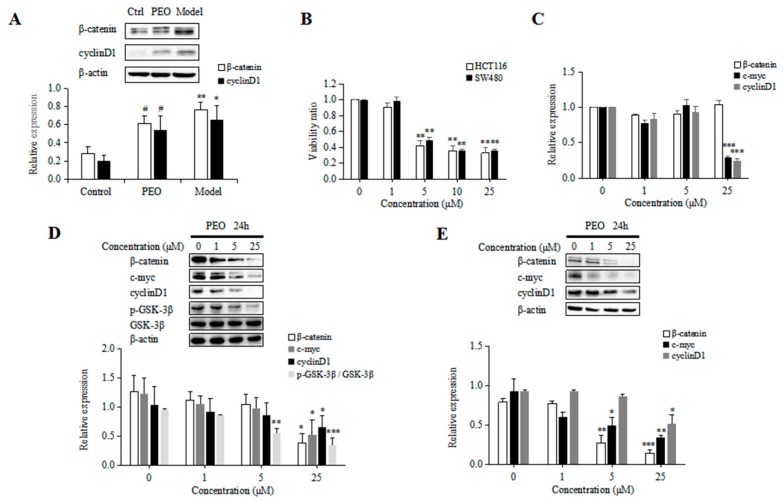
(**A**) PEO inhibits the expression of β-catenin and cyclinD1 in intestinal tissues of animals; (**B**) Anti-proliferative effects of different concentrations of PEO on the growth of HCT116 and SW480 cells; (**C**) PEO inhibits the mRNA expression of c-myc and cyclinD1 in HCT116 cells; (**D**) PEO inhibits the expression of β-catenin, c-myc, cyclinD1 and p-GSK-3β in HCT116 cells; (**E**) PEO inhibits the expression of β-catenin, c-myc and cyclinD1 in SW480 cells. Density values were normalized to levels of β-actin. Data represent the means ± SE of independent experiments. ^#^ *p* < 0.05 relative to model group, * *p* < 0.05, ** *p* < 0.01,*** *p* < 0.001 relative to control group.

**Figure 4:**
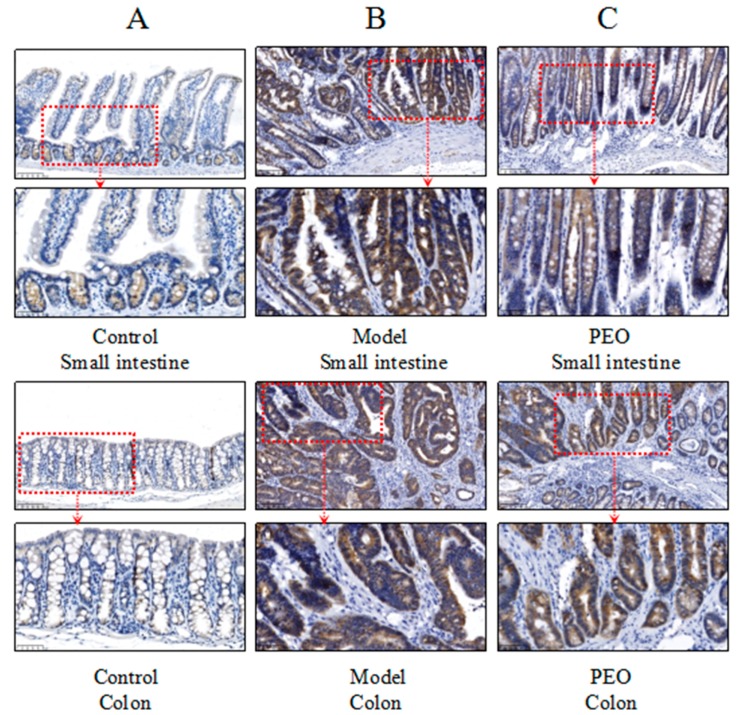
The expression of β-catenin in control group (**A**); model group (**B**); PEO (0.2% diet) group (**C**) was analyzed by immunohistochemistry, bar = 100 µm (20 µm × 5) and bar = 50 µm (10 µm × 5). Samples were scanned by Nano Zoomer 2.0 HT and pictures were obtained from NDP. View 2 (Hamamatsu Photonics K.K., Hamamatsu, Japan).

Activation of the Wnt/β-catenin signaling pathway was shown to be one of the primary drivers of CRC development [21]. In *Apc*^Min/+^ mice, the mutations of Apc gene could activate Wnt/β-catenin signaling by preventing β-catenin degradation, which results in nuclear translocation of stabilized β-catenin and activate target gene transcription. In our study, the results of western blot and immunochemistry showed the expression of β-catenin was upregulated both in the small intestine and colon tissues in the *Apc*^Min/+^ mice, while PEO-treated could significantly reduce its expression (Figure 3A and Figure 4A–C).

In addition, the expression of cyclinD1 was also increased in *Apc*^Min/+^ mice. In contrast, the level of cyclinD1 was significantly decreased after PEO treatment (Figure 3A), indicating PEO might prevents intestinal tumorigenesis in *Apc*^Min/+^ mice via Wnt/β-catenin signaling pathway.

### 2.2. Effects of the PEO in HCT116 and SW480 Cells

#### 2.2.1. Anti-Proliferative Effects of PEO in Colon Cancer Cells

The cytotoxicity of PEO against HCT116 and SW480 cells were measured using MTT assay. Both HCT116 and SW480 cell lines were treated with various concentrations of PEO (1, 5, 10, 25 μM). The cell viability was observed to be reduced in a dose-dependent manner following PEO treatment. The IC_50_ of PEO in SW480 was 3.03 μM, while in HCT116 was only 2.84 μM (Figure 3B), demonstrating the ability of PEO of preventing colon carcinogenesis in vitro.

#### 2.2.2. PEO Decreased the Expression of β-Catenin, CyclinD1, C-myc and P-GSK-3β in HCT116 and SW480 Cells

As PEO could reduce the expressions of β-catenin and cyclinD1 in vivo, we examined the effect of PEO on the expression of these proteins in vitro. As shown in Figure 3D, PEO treatment decreased the protein levels of β-catenin, cyclinD1, c-myc and p-GSK-3β in HCT116 cells at 25 μM. In accordance with this, PEO treatment also down-regulated the protein levels of β-catenin, cyclinD1 and c-myc at 25 μM in SW480 cells (Figure 3E). To further investigate the mediation of β-catenin, cyclinD1, c-myc reduction at protein level or mRNA level, mRNA of β-catenin, cyclinD1, c-myc was quantified in HCT116 cells. In accordance with our western blot results, PEO treatment at 25 μM decreased the mRNA levels of cyclinD1 and c-myc. However, the expression of β-catenin mRNA was not altered upon PEO treatment (Figure 3C).

## 3. Discussion

*O. elatus* has been used for treating neurasthenia, hypoiesis, cardiovascular diseases, diabetes and rheumatism in Russian medicine and Traditional Chinese medicine. However, its bioactive compounds are still not clarified. In this study, we prepared the polyyne-enriched extract from *O. elatus* (PEO) and evaluated its effect on colon cancer. The chromatogram of the PEO, showed FAD and OPD are two main polyynes in PEO extract. The contents of these two compounds are 24.39% and 57.90% in the extract [19]. In addition, UPLC-Q-TOF was used to characteristic the main compounds in PEO extract. As shown in Table S1, 9 peaks were characterized. In order to confirm our hypothesis that polyyne-enriched extract from *O. elatus* is responsible for the anticancer activities of *O. elatus*. *Apc*^Min/+^ mice, a genetically engineered mouse model that has a mutation in the Apc gene were applied. PEO treated significantly improved the body weight changes and stool bleeding score in *Apc*^Min/+^ mice. Compared with the model group, the tumor numbers were significantly reduced after treatment with PEO for 12 weeks. Furthermore, the cell viability was observed to be reduced in a dose-dependent manner following PEO treatment in HCT116 and SW480 cells. In addition, many reports showed the toxicity of *O. elatus* root is very low [13]. No signs of toxicity were observed in mice after acute oral administration at a dose of 50 (g original material/kg) [22]. The safety of the extract of *O. elatus* were examined in 14-day in SD rats. There were no significant changes in body and organ weights when the rats were treated with the extract by gavage at 500, 1000, 2000 mg/kg [23]. In our experiment, the PEO oral administration is about 6 mg per mouse a day. Considering the yield of the extract (67 g extract were obtained from 4 kg crude materials), our dosage is 1.8 (g original material/kg), which is lower than these reported dosages. Taken together, we demonstrated that PEO significantly reduced the progression of carcinogenesis in *Apc*^Min/+^ mice.

The Wnt/β-catenin signaling pathway controls many biological processes, including cell fate determination, cell proliferation and stem cell maintenance [24]. Many researches have been demonstrated the link between Wnt/β-catenin signaling and cancer [25,26]. The key regulatory step involves the phosphorylation, ubiquitination and subsequent degradation of its down-stream effector protein, β-catenin, by a dedicated cytoplasmic destruction complex. The core components in this complex are including Axin, Apc and GSK-3β. Mutations in any of them will result in cancer [27]. Thus, in *Apc*^Min/+^ mice, the mutations of Apc gene could activate Wnt/β-catenin signaling by preventing β-catenin degradation, which results in nuclear translocation of stabilized β-catenin and activate target gene transcription [28]. Consistent with this, in our study, we find the β-catenin is highly accumulated in *Apc*^Min/+^ mice. At the same time, the expression of cyclinD1 was also increased. In contrast, the level of β-catenin and cyclinD1 were significantly decreased after PEO treatment in *Apc*^Min/+^ mice. Meanwhile, PEO treatment decreased the protein levels of β-catenin, cyclinD1, c-myc and p-GSK-3β in HCT116 and SW480 cells at 25 μM. PEO treatment at 25 μM also downregulated mRNA levels of cyclinD1 and c-myc in HCT116 cells. However, the expression of β-catenin mRNA was not altered upon PEO treatment, indicating PEO treatment regulated the expression of β-catenin at the protein level. All these results suggested PEO ameliorates the progression of colon carcinogenesis partly by the regulation of Wnt/β-catenin signaling. In addition, apart from Wnt signaling, many other pathways such as MAPK/PI3K, TGF-β, TP53 also plays an important roles in the carcinogenesis [29]. Further investigation still needed to study the effects of PEO on these pathways. In summary, using the carcinogenesis *Apc*^Min/+^ mouse model, we report that PEO significantly ameliorates tumor initiation and progression. The observed effects were supported by the body weight change and gut tissue histology. Moreover, our works showed that PEO could regulate Wnt/β-catenin signaling in vivo and in vitro, thereby ameliorated colon cancer progression. These findings help us to have a better understanding about the bioactive compounds in *O. elatus* and its anticancer effect action, contributing to its further application in the prevention of colon cancer.

## 4. Materials and Methods

### 4.1. Chemicals, Material and Reagents

The dried *Oplopanax elatus* was collected from natural habitat in Jilin, China, and authenticated by Prof. Wen Xiao-Dong, China Pharmaceutical University, Nanjing, China. Antibodies of c-myc, cyclinD1, β-catenin were purchased from Abcam (Cambridge, UK). Anti-GSK-3β was obtained from Wanleibio (Shenyang, Liaoning, China). Anti-p-GSK-3β was obtained from Santa Cruz Biotechnology (Santa Cruz, CA, USA) and anti-β-actin was purchased from Beyotime (Nanjing, Jiangsu, China).

### 4.2. Preparation of PEO

The PEO was prepared according to our previous paper [19]. Briefly, air-dried roots of *O. elatus* (4 kg) were extracted with refluxing 95% ethanol (28 L) three times for 5 h each. The ethanol extracts were combined and concentrated and the residue was suspended in distilled water. The solution was then partitioned with ethyl acetate. The ethyl acetate fraction was fractionated by column chromatography on silica gel eluted with a gradient of petroleum ether–ethyl acetate (99:1, 95:5). The 95:5 eluent was collected and concentrated under vacuum to give 67 g of residue, which was used as the polyynes extract of *O. elatus.*

To characterize the chemical constituents in PEO, a UPLC-QTOF-MS/MS method was established (Supplement I2). The total ion chromatograms in positive ion modes were displayed in Figure S2A. The UPLC chromatogram of PEO extract recorded at 203 nm is shown in Figure S2B. In Table S1, a total of nine components were detected, and two of those (FAD and OPD) were identified by comparing their retention times, mass accuracy, and fragmentation behaviors with data from the corresponding references [30,31].

**Figure 5:**
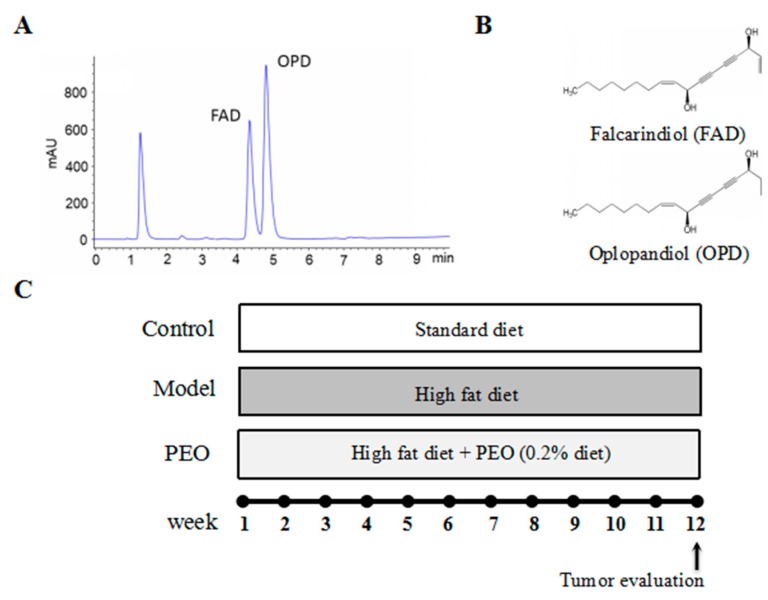
Chemical structures and HPLC analysis of polyynes in PEO extract. HPLC chromatogram of PEO extract recorded at 203 nm is shown in (**A**); Two polyynes in PEO extract were determined: falcarindiol (FAD) and oplopandiol (OPD). The structures of the determined polyynes are shown in (**B**); The percentages of FAD and OPD in PEO extract were 24.39% and 57.90%, respectively. The animal experimental protocol is shown in (**C**).

The determination of FAD and OPD in the PEO was performed by HPLC-ELSD (Figure 5A). The structures of the determined polyynes are shown in Figure 5B. The percentages of facarindiol and oplopandiol were 24.39% and 57.90% [19].

### 4.3. Animal Studies

All procedures described involving animals were performed on the basis of protocols approved by the Institutional Animal Care and Use Committee at the China Pharmaceutical University. Sixteen male C57BL/6J-*Apc*^Min/+^ mice and eight wild-type mice were obtained at 6 weeks of age from the Model Animal Research Center of NanJing University. Animals were maintained in a temperature and humidity controlled facility with 12-h light/dark cycles in the Center for New Drug Safty Evaluation and Research.

All animals were allowed a seven-day acclimation period prior to being randomly assigned to a model (*n* = 8) or PEO group (*n* = 8). The wild-type mice were used as controls (*n* = 8) fed with normal diet (Xietong Organism, Nanjing, China). The model group was fed with high fat diet, while the PEO group was fed with high fat diet supplemented with PEO (0.2% diet, Xietong Organism, Nanjing, China) for 12 weeks. The high-fat diet was composed of protein 13%, carbohydrate 47.6% and fat 39.4% (of total energy, % kcal, Table S2). One mouse ate about 3 g diet a day. Thus, the PEO oral administration is about 6 mg per mouse a day. The experimental protocol is shown in Figure 5C. The animals were weighed daily throughout the experimental period. The minimal stool bleeding score was 0, and the maximal score was 4 (0, none; 1, trace; 2, mild hemoccult; 3, obvious hemoccult; 4, gross bleeding), which was detected by Hemoccult Sensa test strips (Beckman, Brea, CA, USA). At the end of 12 weeks, blood was collected from retinal venous plexus, centrifuged and the serum was harvested and stored at −80 °C. Then, mice were sacrificed by cervical dislocation. The whole intestine was removed immediately after sacrifice and opened longitudinally after washed with ice-cold PBS. The small intestine was cut into three equal parts, namely S1, S2, S3 and the large intestine (C) consisted of cecum and colon. The number, location, and size of visible tumors throughout the intestine were measured to calculate the incidence of adenoma. The total diameter of all the tumors per mouse was named tumor burden. About 1 cm segments from the S1, S2, S3 and C were taken, fixed in 10% phosphate-buffered formalin for 24 h for hematoxylin and eosin staining and immunohistochemistry analyses. The remaining tissues were kept frozen at −80 °C.

### 4.4. Western Blotting

Harvested colon tissues or cultured cell pellets were homogenized and lysed in ice-cold RIPA lysis buffer (P0013B, Beyotime, Nanjing, Jiangsu, China). Protein concentration was determined using a Bicinchoninic Acid Protein Assay kit (P0010S, Beyotime). These protein were electrophoresed in 10% SDS-PAGE gels and transferred to a polyvinylidene difluoride membrane (PVDF, Millipore, Billerica, MA, USA). The membranes were blocked with 5% fat-free milk and incubated with different primary antibodies. The bound antibodies were detected using horseradish peroxidase-conjugated goat anti-rabbit IgG (H + L) (1:1000; KGAA35, Keygen, Nanjing, China) or goat anti-mouse IgG (H + L) (1:2000; KGAA35, Keygen). The PVDF membranes were subsequently subjected to immunoblotting analysis using the enhanced chemiluminescence (ECL, Tanon, Shanghai, China) according to the manufacturer’s protocol on Luminescent Image Analyzer Tanon5200 (Tanon). Finally, blots were quantified using the Tanon Image Analysis System.

### 4.5. Histology and Immunohistochemistry

In an ice bath, small intestine and colon were dissected to obtain tumor and para-tumor tissues. These tissues were fixed in 10% phosphate-buffered formalin, dehydrated, paraffin-embedded, characterized with hematoxylin-eosin staining and analyzed by pathology professionals. The immunohistochemistry were carried out as previously described [32]. The antibodies used in this study were anti-β-catenin for immunohistochemistry.

### 4.6. Cell Culture and Treatment

HCT116 and SW480 human colon cancer cell lines were purchased from the Cell Bank of the Chinese Academy of Sciences (Shanghai, China) and KeyGen Biotech (Nanjing, China), separately. Both lines were maintained at 37 °C in an atmosphere containing 5% CO_2_ in Dulbecco’s modified Eagle’s medium (DMEM, Keygen, Nanjing, China) supplemented with 10% (*v*/*v*) fetal bovine serum (FBS, Gibco, SA). Cells were seeded in 10-cm dish at the density of 1 × 10^5^ cells/dish for 24 h, then treated with either 0.1% DMSO, or different concentrations of PEO for 24 h.

### 4.7. MTT Assay

The cells in the logarithmic phase, between 5 × 10^3^ and 10 × 10^3^ in 100 μL, were seeded in 96-well plate. After 24 h, the cells were treated with various concentrations of PEO ranging from 1 µM to 25 µM. After 24 h of incubation at 37 °C in 5% CO_2_, the supernatant was removed and replaced by 100 μL of 1, 5, 10, 25 μM PEO for 24 h. 20 μL of 3-(4,5-dimethylthiazol-2-yl)-2,5-diphenyltetrazolium bromide (MTT, Sigma, Louis, MO, USA) solution was added to each plate and incubation was performed at 37 °C for 4 h. Medium was removed, and 100 μL dimethyl sulphoxide (DMSO, Hanbang, Jiangsu, China) was added to dissolved the purple crystals of formazan and incubated for 30 min at 37 °C constant temperature oscillator (THZ-C, Taicang, Jiangsu, China). Finally, the optical density (OD) was measured at 490 nm using a Microplate Reader (51119000, Thermo, Waltham, MA, USA). Cell viability was calculated using the following formula:

Cell viability % =[(OD_drug_ − OD_zero_)/(OD_control_ − OD_zero_)] × 100%

### 4.8. Q-PCR

HCT116 cells were seeded at 1 × 10^6^ cells/well in 6-well-plates and cultured overnight. Then, the cells were respectively treated with various concentrations of PEO (0, 1, 5, 25 μM) for 24 h. The total RNA was isolated using Trizol reagent (Thermo) following the supplier’s instruction. The cDNA was synthesized from total RNA using HiScript^®^ II 1st Strand cDNA Synthesis Kit (Vazyme, Nanjing, Jiangsu, China) according to the manufacturer’s instructions. RT-PCR analyses were performed on the Applied Biosystems QuantStudio 3 Real-Time PCR Systems (Thermo) using ChamQ^TM^ SYBR^®^ qPCR Master Mix (Vazyme). The cDNA was amplified with the following primers: c-myc forward, 5′-CTG AGG AGG AAC AAG AAG ATG AG-3′; c-myc reverse, 5′-TGT GAG GAG GTT TGC TGT G-3′; cyclinD1 forward, 5′-GGT TCA ACC CAC AGC TAC TT-3′; cyclinD1 reverse, 5′-CAG CGC TAT TTC CTA CAC CTA TT-3′; β-catenin forward, 5′-CAT CTA CAC AGT TTG ATG CTG CT-3′; β-catenin reverse, 5′-GCA GTT TTG TCA GTT CAG GGA-3′; Glyceraldehyde 3-phosphate dehydrogenase (GAPDH) forward, 5′-GGT GTG AAC CAT GAG AAG TAT GA-3′; GAPDH reverse, 5′-GAG TCC TTC CAC GAT ACC AAA G-3′. The PCR amplifications were performed at 95 °C for 30 s, followed by 40 cycles of thermal cycling at 95 °C for 10 s, and 60 °C for 30 s. All the qPCR analyses were performed in triplicate. Finally, the cycle threshold (C_T_) values were determined for analysis.

### 4.9. Statistics

Statistical analysis was performed using Prism 5.0 software (GraphPad Inc., La Jolla, CA, USA). Each analyzed parameter was expressed as Mean ± SE. The statistical significance of the differences was determined using one-way analysis of variance and Tukey’s multiple comparison test. *p* < 0.05 was considered statistically significant (* *p* < 0.05; ** *p* < 0.01; ***, *p* < 0.001).

## Supplementary Materials

Supplementary materials are available online.
