# Supplementary material for: Polyyne-Enriched Extract from Oplopanax elatus Significantly Ameliorates the Progression of Colon Carcinogenesis in ApcMin/+ Mice
Source: Molecules. 2017 Sep 22;22(10):1593. doi: 10.3390/molecules22101593 (PMC6151504; doi:10.3390/molecules22101593)
Supplement: Supplementary file 1 [file molecules-22-01593-s001.pdf]

Supplementary Materials: Polyyne-enriched extract from *Oplopanax elatus* significantly ameliorates the progression of colon carcinogenesis in *Apc*$^{Min/+}$ mice.

SI1. Results

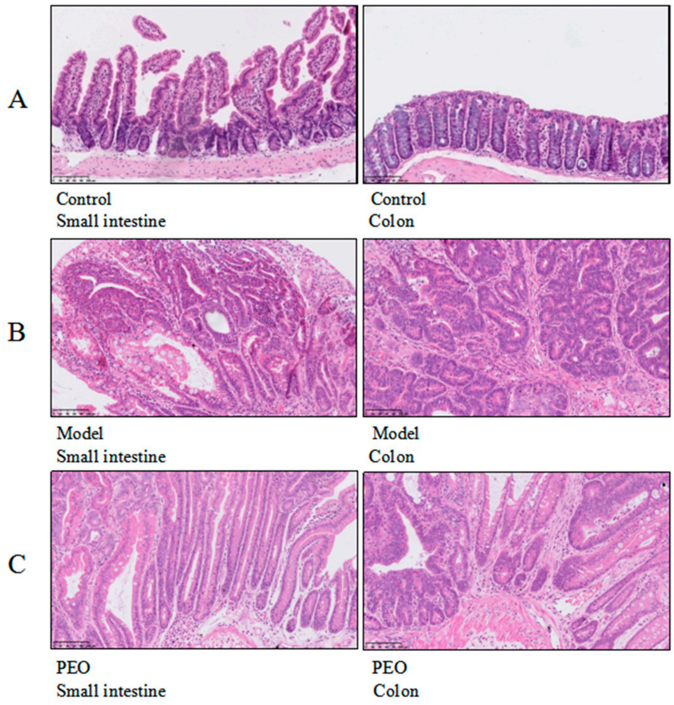

Figure S1. The hematoxylin and eosin staining of the small intestine and colon histological sections, bar = 100 μm (20 μm×5). (A) Normol group, (B) Model group, (C) PEO (0.2% diet) group. Samples were scanned by Nano Zoomer 2.0 HT and pictures were obtained from NDP. view 2 (Hamamatsu Photonics K.K., Hamamatsu, Japan).

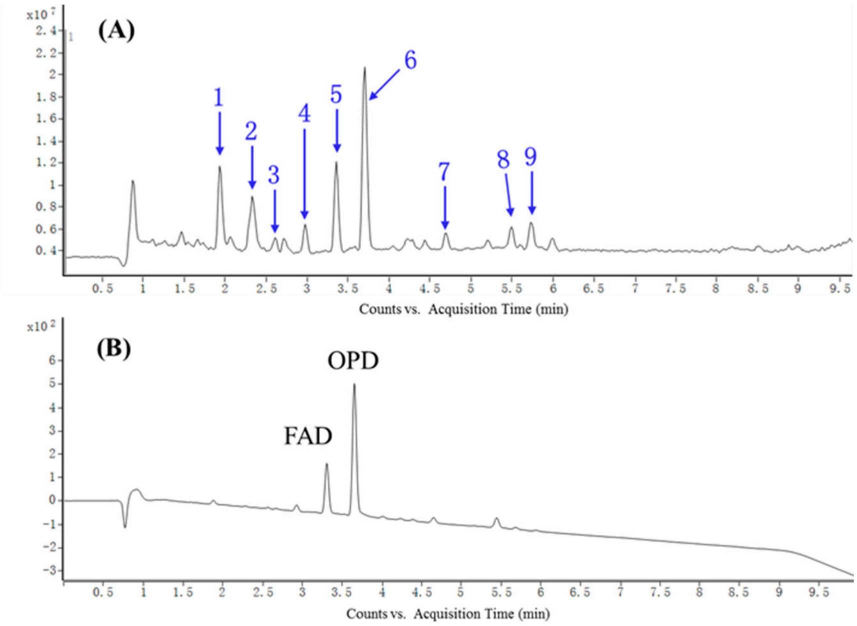

Figure S2. (A) Typical total ion chromatograms of PEO by UPLC-QTOF; (B) UPLC chromatogram of PEO extract recorded at 203nm .

Table S1. Compounds identified in PEO extract UPLC-QTOF-MS analysis in positive ion mode.

NO.	RT(min)	$[M+H]^+$m/z	Compoundformular	Error(ppm)	MS²date	Identification (Ref)
1	1.981	415.2122	$C_{24}H_{30}O_6$	-1.33	397.2003, 295.1164, 281.1388, 135.0808, 119.0869, 147.0644, 91.0542, 81.0333, 69.0332, 57.0340	Unidentified
2	2.262	274.2743	$C_{16}H_{35}NO_2$	-0.65	256.2632, 121.0277, 106.0861, 88.0754, 70.0654, 57.0701	Unidentified
3	2.627	259.1689	$C_{17}H_{22}O_2$	1.66	231.1366, 203.1434, 175.0671, 161.0596, 147.0425, 133.0644, 119.0491, 105.0333, 91.0537, 77.0387, 55.0179	Isofalcarindiolone [1]
4	2.992	275.1636	$C_{17}H_{22}O_3$	1.72	257.1519, 219.1368, 191.0695, 145.0642, 127.0378, 91.0542, 79.0539, 67.0542, 55.0543	Unidentified
5	3.389	225.1637	$C_{17}H_{20}$	-0.29	197.1318, 183.1164, 169.1012, 155.0851, 141.0696, 129.0695, 115.0538, 105.0699, 91.0536, 79.0542, 67.0545, 55.0545	Falcarindiol [2]
6	3.654	227.1795	$C_{17}H_{22}$	-0.01	199.1475, 185.1324, 171.1166, 157.1011, 143.0853, 129.0697, 115.0538, 105.0697, 91.0542, 79.0542, 67.0542, 55.0545	Oplopandiol [2]
7	4.698	318.2066	$C_{19}H_{27}NO_3$	-0.46	276.1941, 191.0692, 135.0439, 107.0485, 77.0386	Unidentified
8	5.477	243.1745	$C_{17}H_{22}O$	0.48	225.1636, 173.0954, 157.0646, 145.1007, 131.0849, 117.0694, 105.0702, 91.0540, 67.0536, 57.0335	Unidentified
9	5.693	261.1846	$C_{17}H_{24}O_2$	1.35	163.0752, 135.0802, 117.0700, 91.0539, 79.0546, 55.0178	Unidentified

Table S2. The high fat diet and AIN76A diet composition.

Diet composition	High fat diet		AIN76A diet	
	% gram	% kcal	% gram	% kcal
Protein	14.3	13	20.3	20.8
Carbohydrate	52.4	47.6	66	67.7
Fat	19.3	39.4	5	11.5
Total		100		100

SI2. Materials and Methods

Methanol and acetonitrile with high-performance liquid chromatography (HPLC)-grade were purchased from Merck Inc (Germeny). Purified water was prepared from a Barnstead Genpure UV/VF (Thermo, USA).

Analyses were performed on an Agilent 1290 liquid chromatographic system (Agilent Technologies, USA) and an Agilent 6545 Quadrupole-Time of flight system (AgilentTechnologies, USA), equipped with an electrospray ionization source operating in positive ion mode. The samples were separated on ACQUITY UPLC® BEH-C_{18} column (2.1mm × 100mm , 1.7 μm , Waters, Ireland). The gradient mobile phase was a mixture of water (A) and acetonitrile (B). The flow rate of 0.3 mL/min at 35 °C was used in linear gradients as follows: 60-80% B (0-8 min), 80-95% B (8-9 min), 95% B (9-10 min). The injection volume was 5 μL.

The following MS parameters were employed: drying gas flow, 8 L/min; source drying gas temperature: 320 °C; nebulizer pressure: 35 psig; capillary voltage: 3.5 kV; Fragmentor: 120V. The MassHunter Qualitative Analysis (Version B.07.00, Agilent Technologies.Inc, USA) was employed for the analyses.

References

[1] Purup, S.; Larsen, E.; Christensen, L.P. Differential effects of falcarinol and related aliphatic C(17)-polyacetylenes on intestinal cell proliferation. *The Journal of Agricultural and Food Chemistry* **2009**, 57, 8290-8296, DOI: 10.1021/jf901503a.

[2] Shao, L.; Nie, M.K.; Chen, M.Y.; Wang, J.; Wang, C.Z.; Huang, W.H.; Yuan, C.S.; Zhou, H.H. Screening and identifying antioxidants from Oplopanax elatus using 2,2'-diphenyl-1-picrylhydrazyl with off-line two-dimensional HPLC coupled with diode array detection and tandem time-of-flight mass spectrometry. *Journal of Separation Science* **2016**, 39, 4269-4280, DOI: 10.1002/jssc.201600838.
